# Clinically suspected central nervous system Richter transformation presenting with progressive psychiatric symptoms: A case report

**DOI:** 10.1002/pcn5.70421

**Published:** 2026-10-19

**Authors:** Tetsuro Ishida, Yusuke Sugama

**Affiliations:** ^1^ Department of Psychiatry and Neurology Sapporo Royal Hospital Sapporo Japan; ^2^ Division of Internal Medicine, Department of Medicine Higashi Sapporo Hospital Sapporo Japan

**Keywords:** central nervous system involvement, chronic lymphocytic leukemia, neuropsychiatric symptoms, psycho‐oncology, Richter transformation

## Abstract

**Background:**

Central nervous system (CNS) involvement associated with Richter transformation (RT) is a rare complication of chronic lymphocytic leukemia (CLL). Neurological manifestations are heterogeneous. To our knowledge, no previous reports have described CNS RT presenting predominantly with psychiatric and behavioral symptoms. We report a clinically suspected case of CNS RT presenting predominantly with progressive psychiatric symptoms in the presence of extensive CNS lesions but without apparent focal neurological deficits.

**Case Presentation:**

A 71‐year‐old man with no psychiatric history developed progressive personality change, irritability, emotional lability, behavioral disinhibition, and sleep–wake cycle disturbance. Gadolinium‐enhanced fluid‐attenuated inversion recovery magnetic resonance imaging demonstrated newly developed multifocal lesions predominantly involving the bilateral temporal lobes. Hematological evaluation established previously unrecognized CLL. Although histopathological confirmation was not obtained, the overall clinical, radiological, and laboratory findings supported CNS involvement associated with clinically suspected RT as the most likely diagnosis. Progressive neuropsychiatric deterioration limited invasive diagnostic procedures and disease‐directed treatment. Supportive and palliative management was provided, and the patient died of aspiration pneumonia 114 days after surgery.

**Conclusion:**

This case demonstrates that clinically suspected CNS RT may present predominantly with progressive psychiatric and behavioral symptoms in the absence of focal neurological deficits. Severe neuropsychiatric symptoms may themselves impede diagnostic confirmation and disease‐directed treatment. When pathological confirmation is not feasible, multidisciplinary discussion and shared decision‐making with the patient and family, together with explicit acknowledgment of diagnostic uncertainty and careful balancing of the benefits and burdens of further investigation and treatment, may support patient‐centered care.

## INTRODUCTION

Chronic lymphocytic leukemia (CLL) is generally an indolent mature B‐cell neoplasm with a highly variable clinical course, and outcomes have substantially improved with modern targeted therapies.^1^ However, a small proportion of patients develop transformation into an aggressive lymphoma, known as Richter transformation (RT), which is associated with a markedly poorer prognosis.^2^

Central nervous system (CNS) involvement in RT is exceptionally rare. Reported manifestations are heterogeneous and include focal neurological deficits, headache, seizures, cognitive impairment, and encephalopathy, whereas psychiatric and behavioral symptoms as the predominant presentation have received limited attention.^3^, ^4^

We report a patient without a previous diagnosis of CLL who developed rapidly progressive psychiatric and behavioral symptoms associated with multifocal CNS lesions and was subsequently diagnosed with CLL with clinically suspected RT. The case illustrates an unusual neuropsychiatric presentation of suspected CNS RT and highlights how severe neuropsychiatric symptoms can themselves constrain diagnostic and therapeutic options.

## CASE PRESENTATION

A 71‐year‐old man with no history of psychiatric illness developed gradually progressive fatigue and changes in personality and behavior approximately 6 months before the current hospitalization. His family noticed increasing irritability, emotional lability, disproportionate anger in response to minor events, and verbally aggressive or socially inappropriate remarks that were inconsistent with his previous personality. These changes were accompanied by reduced motivation and disruption of the sleep–wake cycle. Because of these symptoms, he was evaluated at a neurosurgical department. Brain magnetic resonance imaging (MRI), including T1‐weighted, T2‐weighted, fluid‐attenuated inversion recovery, diffusion‐weighted, and post‐contrast T1‐weighted images, revealed a left parietal extra‐axial mass consistent with a meningioma. Retrospective review revealed no subtle parenchymal abnormalities corresponding to the lesions detected on postoperative Day 28. On Day 0, he underwent surgical resection of the lesion, and histopathological examination confirmed a World Health Organization Grade 1 meningioma. Postoperative examinations revealed no clinically significant abnormalities, and he was discharged.

However, his fatigue and psychiatric and behavioral symptoms did not improve after surgery and instead gradually worsened. On Day 28, because of the persistent and progressive symptoms, follow‐up MRI was performed earlier than initially planned. Gadolinium‐enhanced fluid‐attenuated inversion recovery MRI demonstrated newly developed multifocal lesions predominantly involving the bilateral temporal lobes, with additional scattered lesions in the right parietal region (Figure 1). These abnormalities had not been present on the preoperative MRI. Despite the extensive intracranial abnormalities, neurological examination revealed no apparent focal neurological deficits, and psychiatric and behavioral symptoms remained the predominant clinical manifestations.

**Figure 1:**
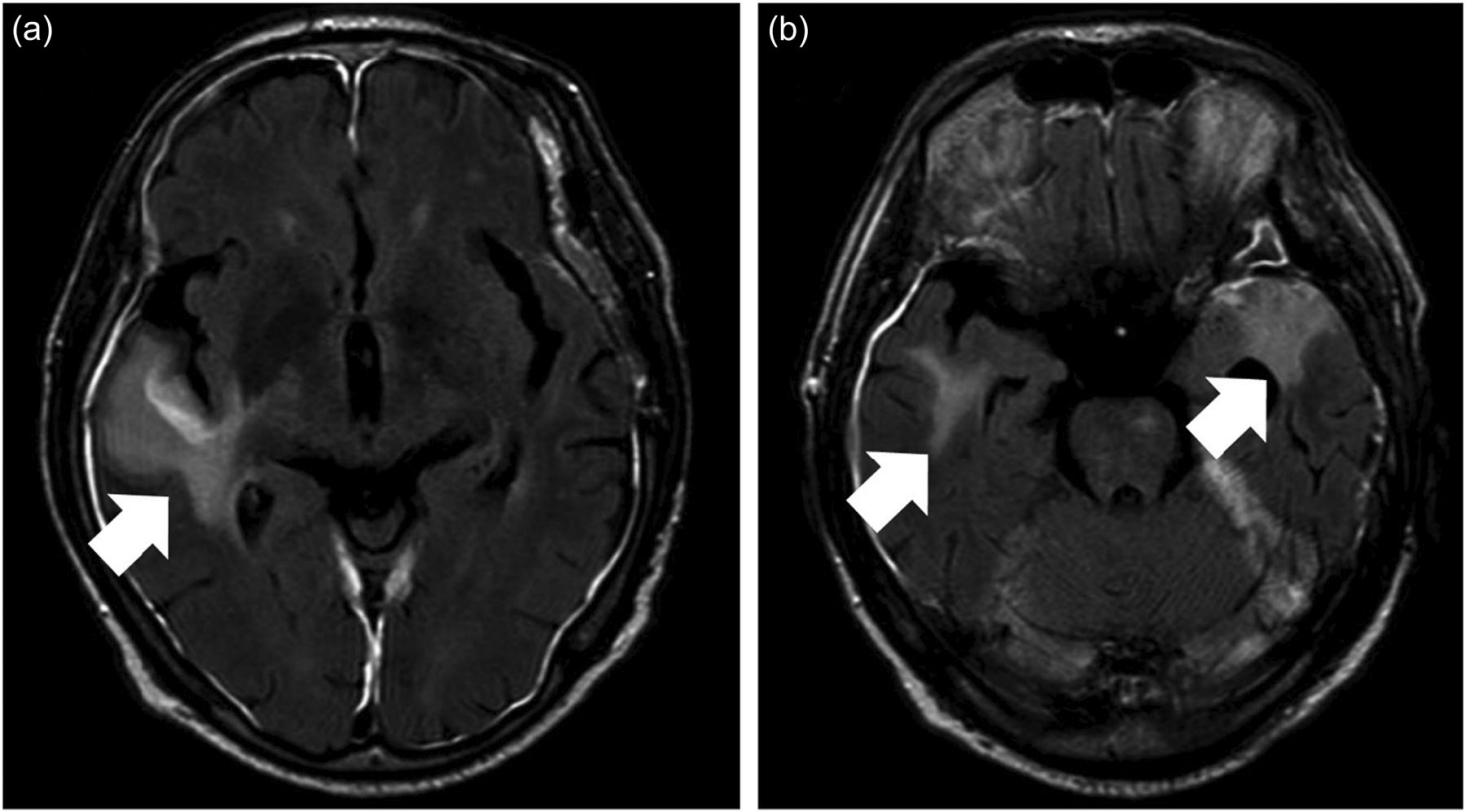
Gadolinium‐enhanced fluid‐attenuated inversion recovery magnetic resonance images obtained on postoperative Day 28. (a) Axial image demonstrating an enhancing lesion predominantly involving the right temporal lobe with surrounding edema (white arrow). (b) A more inferior axial image demonstrating multifocal enhancing lesions involving the bilateral temporal lobes (white arrows).

Subsequent laboratory examination at the neurosurgical department revealed leukocytosis (white blood cell count, 21.17 × 10^9^/L) with an absolute lymphocyte count of 13.00 × 10^9^/L. Peripheral blood examination showed lymphoid cells and smudge cells, prompting consultation with the hematology department. The patient was subsequently referred to the hematology department for further evaluation, including systemic computed tomography and bone marrow examination, and was diagnosed with CLL on Day 43. Systemic imaging showed no significant lymphadenopathy, hepatosplenomegaly, or other malignancy that could account for the CNS lesions.

Cerebrospinal fluid (CSF) examination showed no significant abnormalities, and cytological examination revealed no malignant cells. Serological testing for human immunodeficiency virus, hepatitis B virus, hepatitis C virus, and syphilis was negative, and CSF polymerase chain reaction testing for herpes simplex virus and varicella‐zoster virus was also negative. In view of the newly diagnosed CLL, progressive neuropsychiatric syndrome, newly developed multifocal CNS lesions, and absence of a more convincing alternative diagnosis, CNS involvement associated with clinically suspected RT was considered the most likely diagnosis.

Thereafter, his neuropsychiatric condition deteriorated rapidly. He became largely bedridden and exhibited persistent severe psychomotor agitation, frequently shouting and vigorously moving his arms and legs. Meaningful verbal communication became almost impossible, and psychiatric consultation was initiated. Because reliable oral medication could not be achieved, transdermal blonanserin at 80 mg/day was used for agitation and behavioral dysregulation, with limited benefit.

Histopathological confirmation by brain biopsy was considered but ultimately not pursued. By this stage, the CNS lesions were extensive and multifocal, and his profound behavioral dysregulation and inability to cooperate meant that invasive diagnostic procedures would likely have required substantial physical or pharmacological restraint. No treatment specifically directed at the underlying CLL was administered. Disease‐directed treatment, including corticosteroids and molecularly targeted therapy, as well as radiotherapy, was also considered. However, his persistent severe agitation and inability to cooperate substantially limited the feasibility of safely administering these treatments.

After discussion with his family, they preferred not to pursue invasive diagnostic procedures that were unlikely to lead to curative treatment and would impose additional restraint and distress. The goals of care were therefore shifted toward supportive management focused on relief of distress rather than further invasive diagnostic evaluation or disease‐directed treatment. On Day 85, he was transferred to our hospital for supportive and palliative care. At transfer, his Eastern Cooperative Oncology Group performance status was 4; he was almost entirely bedridden, meaningful verbal communication was severely impaired, and oral intake was markedly compromised.

The attending psychiatrist assessed distress and neuropsychiatric symptoms, supported the patient and family, and coordinated multidisciplinary management of distress and agitation, with a shared focus on comfort and avoidance of burdensome interventions. Because transdermal blonanserin had provided limited benefit, the attending psychiatrist gradually reduced and subsequently discontinued it. After discontinuation, he occasionally raised his voice during potentially painful procedures such as blood sampling but otherwise showed no substantial recurrence of severe agitation and generally appeared calm. He also responded more frequently to verbal prompts, suggesting that reduced agitation was not simply due to declining consciousness; however, a causal relationship with blonanserin discontinuation could not be established. Management thereafter focused on maintaining comfort and relieving distress. Thereafter, his level of consciousness gradually declined, accompanied by increasingly irregular respiration. He subsequently developed aspiration pneumonia. Despite antimicrobial and supportive treatment, his respiratory and general condition progressively deteriorated, and he died on Day 114.

## DISCUSSION

### Neuropsychiatric presentation of CNS Richter transformation

CNS involvement in RT is exceptionally rare, and the available literature has consisted predominantly of individual case reports and small case series.^3^, ^4^ Among these rare cases, CNS RT diagnosed concurrently with previously unrecognized CLL appears to be even more exceptional. Xu et al. reported the first such case, in which CNS RT and CLL were diagnosed concurrently in a patient without evidence of lymphoma outside the CNS.^4^ That patient presented with neurological symptoms, including slurred speech, headache, and hemiparesis.^4^ In contrast, our patient presented predominantly with progressive psychiatric and behavioral symptoms despite extensive multifocal CNS lesions and had no apparent focal neurological deficits. To our knowledge, this represents the first reported case of CNS involvement associated with RT diagnosed concurrently with previously unrecognized CLL in which psychiatric and behavioral symptoms predominated over neurological manifestations.

The predominant involvement of the bilateral temporal lobes may have contributed particularly to the subsequent worsening of the neuropsychiatric manifestations, given the established role of temporolimbic networks in the regulation of emotion and behavior.^5^ However, because the available preoperative MRI sequences showed no corresponding parenchymal abnormalities, there is insufficient evidence to attribute the initial psychiatric and behavioral symptoms to CNS involvement of RT. Although radiologically occult involvement at the time of symptom onset cannot be completely excluded, this possibility remains speculative. Importantly, CLL had not been diagnosed when the neuropsychiatric symptoms first emerged. Thus, in patients with otherwise unexplained progressive neuropsychiatric symptoms and newly developing multifocal brain lesions, CNS involvement associated with an underlying hematological malignancy should be considered when hematological abnormalities are present, even if they are initially nonspecific or have not previously led to the recognition of a hematological disorder.

### Neuropsychiatric symptoms as barriers to diagnosis and treatment

A second important feature of this case was that the neuropsychiatric symptoms were not merely manifestations of CNS disease but became determinants of what diagnostic and therapeutic interventions remained feasible. Following the detection of the CNS lesions, the patient's behavioral dysregulation progressed rapidly to severe psychomotor agitation, impaired communication, and inability to cooperate with medical procedures.

In our patient, severe agitation and inability to cooperate transformed the neuropsychiatric syndrome from a clinical manifestation into a practical barrier to care. Diagnostic confirmation and disease‐directed treatment could not be pursued without imposing substantial restraint and distress. Thus, neuropsychiatric symptoms arising from CNS disease may directly determine which diagnostic and therapeutic interventions remain realistically feasible.

More broadly, pre‐existing psychiatric morbidity has been associated with disparities across the cancer care pathway, including reported delays in cancer diagnosis^6^ and lower receipt of guideline‐recommended cancer treatment.^7^ These studies were not specific to hematological malignancies or CNS involvement and predominantly concerned patients with pre‐existing psychiatric disorders. Their findings therefore cannot be directly extrapolated to neuropsychiatric symptoms caused by an organic CNS disorder. Nevertheless, the present case illustrates a related but distinct clinical problem: severe neuropsychiatric symptoms arising from the disease itself may directly alter the feasibility of cancer diagnosis and treatment. Early recognition and management of such symptoms may therefore be important not only for reducing distress but also, when possible, for preserving opportunities for diagnostic evaluation and disease‐directed therapy.

### Diagnostic uncertainty

The principal limitation of this case is the absence of histopathological confirmation of RT involving the CNS. The diagnosis therefore remained clinical, based on the newly diagnosed CLL, rapidly emerging multifocal CNS lesions, subsequent clinical deterioration, and lack of a more convincing infectious or malignant alternative.

Importantly, negative CSF cytology does not exclude CNS lymphoma. Conventional CSF cytology has limited sensitivity for CNS lymphoma, and a recent systematic review of 144 studies involving 9493 patients with primary CNS lymphoma found a pooled detection rate of only 17% for cytology and 20% for flow cytometry.^8^ Earlier diagnostic reviews likewise reported low sensitivity of CSF cytology and emphasized that tissue biopsy remains important when definitive diagnosis is required. Accordingly, the absence of malignant cells in the CSF in our patient does not, by itself, substantially exclude CNS lymphomatous involvement. Although these data derive primarily from patients with primary CNS lymphoma rather than CNS RT, they demonstrate the limited ability of negative CSF findings to exclude CNS lymphomatous disease.^9^

Nevertheless, without tissue confirmation, alternative diagnoses cannot be completely excluded, and the diagnosis of CNS RT should therefore remain explicitly qualified as clinically suspected.

### Shared decision‐making

This diagnostic uncertainty was inseparable from treatment decision‐making. In this case, the value of obtaining greater diagnostic certainty had to be weighed against the substantial burden of further investigation and the limited likelihood that its results would enable feasible disease‐directed treatment. Given the patient's rapidly deteriorating condition, the decision to forgo invasive confirmation and prioritize relief of distress reflected a balance between diagnostic benefit and patient burden.

More broadly, diagnostic uncertainty is often unavoidable in serious illness, particularly when further investigation carries substantial burden and may be unlikely to meaningfully alter management. Shared decision‐making in such circumstances may therefore involve not eliminating uncertainty at all costs, but explicitly acknowledging and communicating what remains uncertain while weighing the expected benefits and burdens of available options.^10^, ^11^

Consultation psychiatrists may have a particular role when neuropsychiatric symptoms themselves complicate this process by impairing communication or procedural cooperation. In the present case, after the patient's transfer for palliative care, the attending psychiatrist assessed distress and neuropsychiatric symptoms, adjusted psychotropic treatment, supported the patient and family, and coordinated with the multidisciplinary team to maintain a shared focus on comfort‐oriented care. More broadly, in addition to symptom management, psychiatrists may help facilitate multidisciplinary decision‐making with patients and families when diagnostic certainty, treatment feasibility, and patient burden must be considered together.

## CONCLUSION

CNS involvement associated with RT should be considered in the differential diagnosis of rapidly progressive neuropsychiatric symptoms with multifocal brain lesions, even in patients without known CLL. Severe neuropsychiatric symptoms may not only reflect underlying CNS disease but also restrict diagnostic and therapeutic options. When pathological confirmation is not feasible, acknowledging diagnostic uncertainty and balancing the benefits and burdens of further investigation through multidisciplinary shared decision‐making may support patient‐centered care.

## AUTHOR CONTRIBUTIONS

Tetsuro Ishida was the patient's attending physician during palliative care and contributed to the literature review and manuscript preparation. Yusuke Sugama contributed to the literature review and critically revised the manuscript. Both authors approved the final version of the manuscript.

## CONFLICT OF INTEREST STATEMENT

Tetsuro Ishida has received lecture fees from Otsuka Pharmaceutical Co., Ltd., Shionogi & Co., Ltd., and Sumitomo Pharma Co., Ltd. These relationships are unrelated to the present work.

## ETHICS APPROVAL STATEMENT

Ethical approval was not required for this single case report in accordance with institutional policy. This report was prepared in accordance with the principles of the Declaration of Helsinki.

## PATIENT CONSENT STATEMENT

Written informed consent for publication of this case report and the accompanying images was obtained from the patient's legal representative.

## CLINICAL TRIAL REGISTRATION

Not applicable.

## Data Availability

Data sharing is not applicable to this article because no datasets were generated or analyzed during the current report.

## References

[1] Hallek M . Chronic lymphocytic leukemia: 2025 update on the epidemiology, pathogenesis, diagnosis, and therapy. Am J Hematol. 2025;100:450–480. 10.1002/ajh.27546 PMC11803567 39871707

[2] Hampel PJ , Rabe KG , Wang Y , Hwang SR , Kenderian SS , Muchtar E , et al. Incidence of Richter transformation of chronic lymphocytic leukemia/small lymphocytic lymphoma in the targeted therapy era. Leukemia. 2025;39:503–507. 10.1038/s41375-024-02492-4 39658628

[3] Baccon D , Atallah‐Yunes SA , Hampel PJ , Roeker LE , Kenderian SS , Muchtar E , et al. Central nervous system involvement in patients with Richter transformation of chronic lymphocytic leukemia (CLL): the Mayo Clinic experience. Blood Cancer J. 2026;16:7. 10.1038/s41408-025-01446-y PMC12779626 41501021

[4] Xu L , Song JC , Sun XH , Gao ZF , Lv L , Zhu J . Richter's syndrome of the central nervous system diagnosed concurrently with chronic lymphocytic leukaemia: a case report and literature review. Medicine. 2018;97:e12701. 10.1097/MD.0000000000012701 PMC6203471 30313065

[5] Trimble MR , Mendez MF , Cummings JL . Neuropsychiatric symptoms from the temporolimbic lobes. J Neuropsychiatry Clin Neurosci. 1997;9:429–438. 10.1176/jnp.9.3.429 9276844

[6] Gorodensky JH , Davis L , Griffiths R , Ayonrinde O , Webber C , Hanna TP , et al. Inequities in the time to colon cancer diagnosis among individuals with severe psychiatric illness. Cancer Med. 2025;14:e70623. 10.1002/cam4.70623 PMC11831619 39960259

[7] Wang Y‐H , Aggarwal A , Stewart R , Davies EA . Impact of pre‐existing mental health disorders on the receipt of guideline recommended cancer treatments: a systematic review. Psycho‐Oncology. 2023;32:307–330. 10.1002/pon.6081 36588188

[8] van Rooij J , Snijders T , Bhande P , Seute T , Minnema M , Wessels P . The diagnostic yield of cerebrospinal fluid analysis for the diagnosis of primary central nervous system lymphoma: a systematic review. Cancers. 2025;17:2352. 10.3390/cancers17142352 PMC12293505 40723236

[9] Scott BJ , Douglas VC , Tihan T , Rubenstein JL , Josephson SA . A systematic approach to the diagnosis of suspected central nervous system lymphoma. JAMA Neurol. 2013;70:311–319. 10.1001/jamaneurol.2013.606 PMC4135394 23319132

[10] Pieterse AH , Huijgens FL , Vromans RD , Hillen MA . The interplay between shared decision making and uncertainty—a conceptual analysis and empirical illustration. Patient Educ Couns. 2026;147:109515. 10.1016/j.pec.2026.109515 41720067

[11] Chen R , Mourhli J , Bowers B , Koffman J , Barclay S , Spathis A , et al. What should we aim for when addressing uncertainty from serious illness? A stakeholder focus group study. J Gen Intern Med. 2026;41:2725–2733. 10.1007/s11606-026-10364-z PMC13421509 41949805

